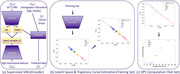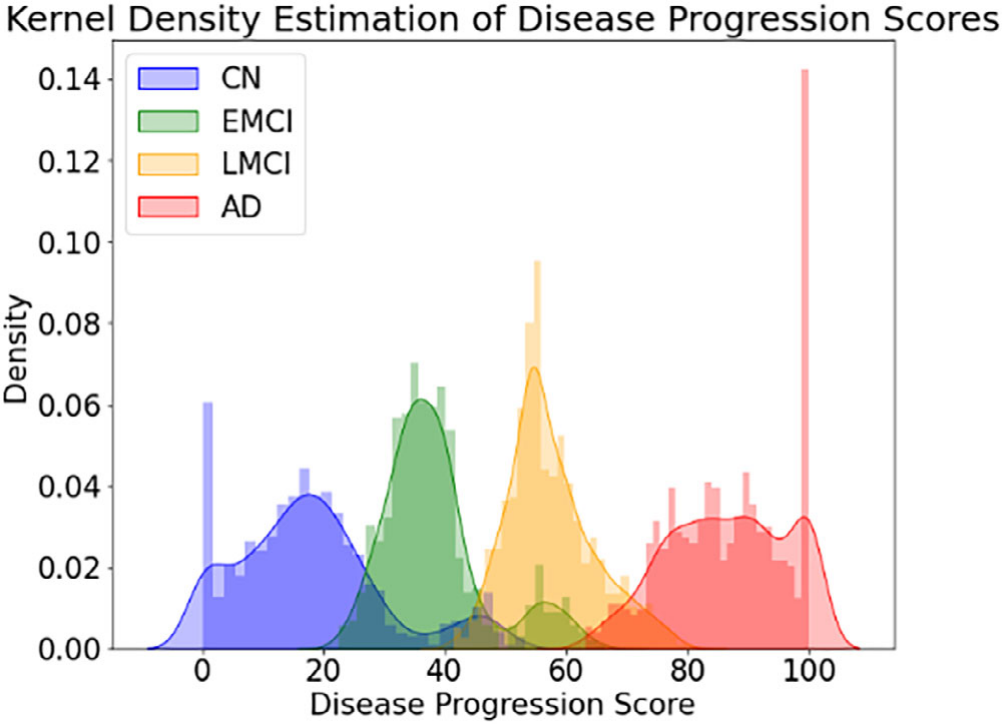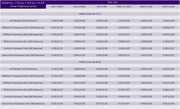# Estimating Alzheimer's Disease Progression Score Using Machine Learning on FreeSurfer‐Derived MRI Gray Matter Volumes

**DOI:** 10.1002/alz.087163

**Published:** 2025-01-09

**Authors:** Junhyoun Sung, Dean Shibata, Kwun Chuen Gary Chan, Lan Shui, David R. Haynor

**Affiliations:** ^1^ University of Washington, Seattle, WA USA; ^2^ National Alzheimer's Coordinating Center, University of Washington, Seattle, WA USA; ^3^ The University of Texas MD Anderson Cancer Center, Houston, TX USA

## Abstract

**Background:**

The early diagnosis and monitoring of Alzheimer's disease (AD) presents a significant challenge due to its heterogeneous nature, which includes variability in cognitive symptoms, diagnostic test results, and progression rates. This study aims to enhance the understanding of AD progression by integrating neuroimaging metrics with demographic data using a novel machine learning technique.

**Method:**

We used supervised Variational Autoencoders (VAEs), a generative AI method, to analyze high‐dimensional neuroimaging data for AD progression, incorporating age and gender as covariates. We used two non‐overlapping datasets: 257 samples from ADNI3 and 676 from ADNI2, analyzing 68 cortical and 48 subcortical grey matter volumes extracted from their MRI 3D T1 images. The VAE model aimed to minimize reconstruction error (MSE), Kullback‐Leibler divergence, and classification error, and to estimate latent variables for each subject. A Disease Progression Score (DPS) was calculated by encoding the extracted imaging features into a latent space and projecting test data onto a trajectory curve. The study used stratified sampling for robustness and assessed the model's performance using the area under the ROC curve (AUC), and correlated the mean DPS with cognitive assessment scores by applying Kendall’s Tau.

**Result:**

The VAE model demonstrated excellent discriminative power in classifying AD progression stages, with ROC AUC values near 1, particularly when using all 116 features. Cortical volumes were more predictive than subcortical volumes. The mean DPS showed a statistically significant correlation with cognitive assessments (p<0.01), with Kendall's Tau values of 0.66 for CDR‐SB, ‐0.45 for MMSE, and ‐0.49 for MoCA, indicating its validity as a quantitative biomarker for cognitive decline in AD.

**Conclusion:**

Supervised VAEs can effectively model the progression of Alzheimer’s disease brain atrophy on MRI and could potentially serve as imaging biomarkers in clinical trials. The strong correlation between the DPS and cognitive assessments highlights the potential of supervised VAEs to provide a quantifiable measure of AD severity, which would be useful for clinical assessments and for objectively monitoring disease progression. The study's methodology and findings contribute to computational neuroscience and offer a foundation for future research in early detection and personalized treatment strategies for Alzheimer's disease.